# Epicardial Adipose Tissue Thickness Correlates with the Presence and Severity of Angiographic Coronary Artery Disease in Stable Patients with Chest Pain

**DOI:** 10.1371/journal.pone.0110005

**Published:** 2014-10-21

**Authors:** Fabien A. Picard, Pascal Gueret, Jean-Pierre Laissy, Stéphane Champagne, Florence Leclercq, Didier Carrié, Jean-Michel Juliard, Patrick Henry, Ralph Niarra, Gilles Chatellier, Philippe Gabriel Steg

**Affiliations:** 1 Cardiology Department, Assistance Publique-Hôpitaux de Paris, Hôpital Bichat, Université Paris-Diderot, Sorbonne-Paris Cité, Paris, France; 2 Cardiology Department, Assistance Publique-Hôpitaux de Paris, Hôpital Henri Mondor, Université Paris-Est-Créteil, Créteil, France; 3 Radiology Department, Assistance Publique-Hôpitaux de Paris, Hôpital Bichat, Université Paris-Diderot, Sorbonne-Paris Cité, Paris, France; 4 Département Hospitalo-Universitaire FIRE, INSERM U-1148, Assistance Publique-Hôpitaux de Paris, Hôpital Bichat, Université Paris-Diderot, Sorbonne-Paris Cité, Paris, France; 5 Cardiology Department, Centre Hospitalier Universitaire Arnaud de Villeneuve, Montpellier, France; 6 Cardiology Department, Centre Hospitalier Universitaire de Rangueil, Toulouse, France; 7 Cardiology Department, Assistance Publique-Hôpitaux de Paris, Hôpital Lariboisière, Université Paris-Diderot, Sorbonne-Paris Cité, Paris, France; 8 Epidemiology and Clinical Research Unit, Assistance Publique-Hôpitaux de Paris, Hôpital Européen Georges-Pompidou, Paris, France; 9 Unité INSERM Centre D'investigation Épidémiologique 4, Paris, France; 10 Université Paris Descartes, Sorbonne Paris Cité, Faculté de Médecine, Paris, France; 11 NHLI Imperial College, ICMS Royal Brompton Hospital, London, United Kingdom; Children's National Medical Center, Washington, United States of America

## Abstract

**Objective:**

Epicardial adipose tissue (EAT) is suggested to correlate with metabolic risk factors and to promote plaque development in the coronary arteries. We sought to determine whether EAT thickness was associated or not with the presence and extent of angiographic coronary artery disease (CAD).

**Methods:**

We measured epicardial fat thickness by computed tomography and assessed the presence and extent of CAD by coronary angiography in participants from the prospective EVASCAN study. The association of EAT thickness with cardiovascular risk factors, coronary artery calcification scoring and angiographic CAD was assessed using multivariate regression analysis.

**Results:**

Of 970 patients (age 60.9 years, 71% male), 75% (n = 731) had CAD. Patients with angiographic CAD had thicker EAT on the left ventricle lateral wall when compared with patients without CAD (2.74±2.4 mm vs. 2.08±2.1 mm; p = 0.0001). The adjusted odds ratio (OR) for a patient with a LVLW EAT value ≥2.8 mm to have CAD was OR = 1.46 [1.03–2.08], p = 0.0326 after adjusting for risk factors. EAT also correlated with the number of diseased vessels (p = 0.0001 for trend). By receiver operating characteristic curve analysis, an EAT value ≥2.8 mm best predicted the presence of>50% diameter coronary artery stenosis, with a sensitivity and specificity of 46.1% and 66.5% respectively (AUC:0.58). Coronary artery calcium scoring had an AUC of 0.76.

**Conclusion:**

Although left ventricle lateral wall EAT thickness correlated with the presence and extent of angiographic CAD, it has a low performance for the diagnosis of CAD.

## Introduction

Epicardial adipose tissue (EAT) is a visceral adipose tissue surrounding the heart and the coronary arteries. Because of its endocrine and paracrine activity, secreting pro-inflammatory and anti-inflammatory cytokines and chemokines, it has been suggested to influence coronary atherosclerosis development [1]–[5]. The EVASCAN (EVAluation of CT SCANner) study [6] was recently performed to establish the diagnostic accuracy of computed tomography coronary angiography compared to conventional coronary angiography (CA) in a population of symptomatic patients with a clinical indication for anatomical coronary imaging.

Using EVASCAN data, which provided precise assessment of coronary artery disease by CA and the measure of EAT by cardiac computed tomography (CT) in a large cohort of patients, the current analysis was performed to clarify a possible link between EAT and CAD. Our hypothesis was that EAT, as measured by CT scanner, was associated with the presence and extent of angiographic CAD.

## Methods

### Study population

We used EVASCAN data, which provided precise assessment of coronary artery disease by CA and the measure of EAT by cardiac CT to perform this study. Therefore, EVASCAN inclusion criteria were used. EVASCAN was a prospective study of correlation between CT angiography and conventional angiography in stable adults with chest pain referred for non-emergent invasive CA. Eligible patients were ≥18 years old with known or suspected CAD, able to undergo cardiac CT first, then CA within four days. The main exclusion criteria were: unstable clinical status, serum creatinine>150 µmol/L, atrial fibrillation, pregnancy and lactation. The protocol of this study complies with the Declaration of Helsinki, was approved by the institutional review board of Paris VI University and written informed consent was obtained from each patient. Classical CAD risk factors were recorded. The clinical characteristics of the patients are summarized in Table 1.

**Table 1:** Population characteristics and comparison of the presence of significant angiographic coronary artery disease.

	All patients (n = 970)	Presence of significant coronary artery disease		
		No	Yes	
Characteristics		N = 239	N = 731	P
Men, n (%)	689 (71.03%)	122 (51.05%)	567 (77.56%)	**<0.0001**
Age (yrs), mean ± SD	60.85±11.36	57.19±12.29	62.05±10.77	**<0.0001**
BMI (kg/m2), mean ± SD	27.38±4.52	27.09±5.02	27.48±4.34	0.2614
Waist circumference (cm); mean ± SD	98.75±13.31	97.33±15.40	99.23±12.51	0.1601
Current smoker, n (%)	244 (25.15%)	59 (24.69%)	185 (25.31%)	0.8475
Diabetes, n (%)	244 (25.15%)	42 (17.57%)	181 (24.76%)	**0.0218**
Hypertension, n (%)	501 (51.65%)	95 (39.75%)	406 (55.54%)	**<0.0001**
Dyslipidemia, n (%)	427 (44.02%)	86 (35.98%)	341 (46.65%)	**0.0039**
Familial history of CAD; n (%)	660 (68.04%)	159 (66.53%)	501 (68.54%)	0.5631
Metabolic syndrome, n (%)	62 (6.39%)	11 (4.60%)	51 (6.98%)	0.1927
Total cholesterol (g/L), mean ± SD	2.09±1.04	2.09±0.92	2.09±1.07	0.9546
LDL cholesterol (g/L), mean ± SD	1.24±0.73	1.25±0.64	1.24±0.75	0.9163
HDL cholesterol (g/L), mean ± SD	0.54±0.29	0.59±0.32	0.53±0.28	**0.0255**
Calcium scoring, median [IQR]	15.50 [0.00; 331.00]	0.00 [0.00; 9.00]	82.00 [6.00; 626.00]	**<0.0001**
Calcium scoring; mean ± SD	379.72±840.59	72.43±275.58	539.28±980.08	**<0.0001**
LVLW EAT thickness (mm); mean ± SD	2.58±2.31	2.08±2.05	2.74±2.37	**0.0001**
RVLW EAT thickness (mm); mean ± SD	5.38±3.05	4.77±2.73	5.58±3.13	**0.0004**
LVLW EAT thickness ≥2.8 mm, n (%)	427 (44.02%)	83 (34.73%)	344 (47.06%)	**0.0009**
RVLW EAT thickness ≥5.3 mm, n (%)	405 (41.75%)	77 (32.22%)	328 (44.87%)	**0.0005**

BMI  =  body mass index; CAD  =  coronary artery disease; LDL  =  low-density lipoprotein; HDL  =  high-density lipoprotein; LVLW  =  left ventricle lateral wall; EAT  =  epicardial adipose tissue; RVLW  =  right ventricle lateral wall.

### Cardiac CT and coronary angiography protocol

Patients underwent cardiac CT (684 (70.5%) patients had 64 row CT and 286 patients (29.5%) had 16 to 40 row CT) followed by conventional CA. Cardiac CT was performed using a standardized, optimized protocol for each system. All patients were in sinus rythm before cardiac CT. A beta-blocker was recommended if heart rate was>65 beats/minute.

Patients first underwent an unenhanced prospective ECG-gated acquisition for calcium scoring (Agatston score) and then a retrospective ECG-gated contrast-enhanced acquisition to explore the coronary tree and EAT. Scanning parameters varied according to the system used. Current intensity modulation was systematically applied to reduce radiation during systolic phases. The effective dose of the non-enhanced scan and the computed tomography coronary angiography was estimated from the product of the dose–length and a conversion coefficient (k = 0.017mSv/[mGy × cm]) for the chest as the investigated anatomic region [7].

A systematic reconstruction of the cardiac phases encompassing the RR interval (in 10% increments) was performed in all patients. Data were uploaded to dedicated workstations (Advantage Windows, GE; Brilliance, Philips; Leonardo, Siemens; Vitrea, Toshiba).

Conventional CA was performed using standard techniques via a femoral or radial approach [8]. All studies were performed using digital equipment. Multiple projections were obtained as deemed necessary by the angiographer.

### Cardiac CT and CA interpretation

Cardiac CT and CA were analyzed visually in separate core laboratories in a blinded manner by experienced readers unaware of the patient's clinical information or the results of the other imaging technique.

For cardiac CT, EAT was defined as the adipose tissue between the surface of the heart and the visceral epicardium surrounding the 3 main coronary arteries. To determine EAT values, epicardial fat maximal thickness was measured at two different locations: on the left ventricle lateral free wall (LVLW) at the base of the ventricles in short-axis view and on the right ventricle lateral free wall (RVLW) at the base of the ventricles in short-axis view. Maximal thickness was measured from the visceral epicardium to the outside of the myocardium, and perpendicular to the surface of the heart. Maximal LVLW EAT thickness and maximal RVLW EAT thickness were used for analysis. EAT thickness was not measured at other sites (right atrioventricular groove, left atrioventricular groove, interventricular grooves…).

For CA, coronary arteries were scored using the American Heart Association coronary artery classification [9]. Each coronary segment was visually graded as: individually assessable or not; normal; non-significant stenosis (<50%); stenosis ≥50%; or total occlusion. In case of multiple lesions in a given segment or artery, the worst lesion was considered.

### Baseline clinical and biological assessment

Baseline measurements were obtained for the following: total cholesterol, high-density lipoprotein cholesterol, low-density lipoprotein cholesterol, triglycerides; Body mass index (BMI) and waist circumference. Presence of high blood pressure, diabetes, coronary heredity and current or former smoker was recorded.

Biological samples were processed through local laboratories of each hospital.

### Statistical analysis

Descriptive data are presented as frequencies and percentages. Continuous data are expressed as mean ± SD or median [IQR] according to the distribution of the parameter. Risk factors were compared between patients with and without CAD. Student's t test for independent groups was used to compare continuous variables, and chi-square test was used for categorical variables. The relation between cardiovascular risk factors and presence of CAD was assessed with univariate and multivariate logistic regression analysis. This relation was expressed by odds ratios (95% CIs). To determine the best cut-off of EAT thickness and calcium scoring, the Youden index (sensitivity + specificity –1) was calculated using the receiver operating characteristic (ROC) curve. The main aim of the Youden index is to find the best cut-off from the ROC curve, maximizing the difference between true positive rate (Sensitivity) and false positive rate (1-specificity). To determine the intra-observer variability of EAT measurements, one observer repeated the EAT data analysis for 25 randomly chosen CT-scanners on different days. Two observers measuring the already recorded image estimated inter-observer variability. The agreement for both intra- and inter-observer variability was evaluated by intra- and inter-class correlation coefficients (ICC). The ICC value is the ratio of the between-subject variance to the sum of the between-subject variance and the within-subject variance. The smaller the within-subject variance, the better the agreement and the higher the ICC. All statistical analyses were performed using SAS system version 9.2 (SAS Institute, Cary, NC, USA). A 2-tailed p-value <0.05 was considered statistically significant.

## Results

### Patients characteristics

Between June 2006 and June 2008, 40 centres prospectively enrolled 1254 patients; data from 970 were used in the present analysis. The patients excluded from the analysis had either incomplete, poor quality or missing Cardiac CT or CA, missing data for EAT thickness or withdrew consent. The baseline characteristics are shown in Table 1. The mean age was 61±11 years and 71% were male. The main cardiovascular risk factors were familial history of CAD in 68% of patients, treated hypertension in 52% of the patients, dyslipidaemia in 44% of the patients, current smoking in 25% of the patients and diabetes in 25% of the patients. Mean BMI was 27.4±4.5 kg/m2 and waist circumference was 99±13 cm.

EAT thickness measured by cardiac CT for the entire study sample was 2.58±2.31 mm for the LVLW and 5.38±3.05 mm for the RVLW.

The reproducibility of EAT measurements in our study was high both in terms of intra- and inter-observer variability (correlation coefficients: 0.89 and 0.74 respectively).

### Epicardial fat thickness and presence of angiographic CAD

Seven hundred and thirty one patients (75%) were found to have significant CAD on coronary angiography. Their clinical and CT findings were compared to the remaining two hundred and thirty nine patients without significant CAD.

Baseline characteristics of the 2 groups (with or without CAD) are listed in Table 1.

Patients with angiographic evidence of CAD were more frequently males, older, with a higher prevalence of conventional risk factors (diabetes, hypertension, dyslipidaemia) although the prevalence of current smoking, the average BMI, waist circumference and metabolic syndrome were not different from patients without CAD. Total cholesterol and low-density lipoprotein cholesterol were similar among groups (probably because of a high prevalence of statin use). However, HDL levels were lower.

Patients with angiographic CAD had thicker EAT on both left and right ventricle lateral walls, when compared with patients without CAD (2.74±2.4 mm vs. 2.08±2.1 mm; p = 0.0001 for LVLW and 5.58±3.1 mm vs. 4.77±2.7 mm; p = 0.0004 for RVLW). Calcium scoring mean was also higher in patients with angiographic CAD (539.28±980.08 vs. 72.43±275.58; p<0.0001). (Table 1).

The odds ratio (OR) for a patient with a LVLW EAT value ≥2.8 mm to have CAD was 1.67 (95%CI 1.23 to 2.26). This relation remained significant after adjusting for CAD risk factors (OR = 1.46 [1.03–2.08], p = 0.0326) (Table 2). Unlike LVLW EAT; the OR for a patient with a RVLW EAT value ≥5.3 mm to have CAD did not remain significant after adjusting for CAD risk factors (OR = 1.38 [0.97–1.98], p = 0.0757).

**Table 2:** Logistic regression analysis of the association between risk factors and presence of significant angiographic CAD.

	Univariate analysis			Multivariate analysis		
	OR	[95%CI]	P	OR	[95%CI]	P
Men	3.32	[2.44–4.51]	**<0.0001**	4.25	[2.98–6.05]	**<0.0001**
Age	1.04	[1.02–1.05]	**<0.0001**	1.04	[1.03–1.06]	**<0.0001**
BMI	1.02	[0.99–1.05]	0.2614	1.01	[0.98–1.05]	0.4632
Current smoker (yes vs. no)	1.03	[0.74–1.45]	0.8482	1.40	[0.92–2.14]	0.1139
Diabetes (yes vs. no)	1.54	[1.06–2.24]	**0.0226**	1.25	[0.79–1.98]	0.3469
Hypertension (yes vs. no)	1.90	[1.41–2.56]	**<0.0001**	1.89	[1.31–2.74]	**0.0007**
Dyslipidemia (yes vs. no)	1.56	[1.15–2.10]	**0.0041**	1.72	[1.22–2.42]	**0.0021**
Family history of CAD (yes vs. no)	1.10	[0.80–1.50]	0.5632	1.07	[0.75–1.53]	0.6985
Metabolic syndrome (yes vs. no)	1.55	[0.80–3.03]	0.1965	0.87	[0.39–1.92]	0.7241
LVLW EAT (≥2.8 vs. <2.8 mm)	1.67	[1.23–2.26]	**0.0009**	1.46	[1.03–2.08]	**0.0326**

OR  =  odds ratio; CI  =  confidence interval; other abbreviations as in Table 1.

**Figure 1:**
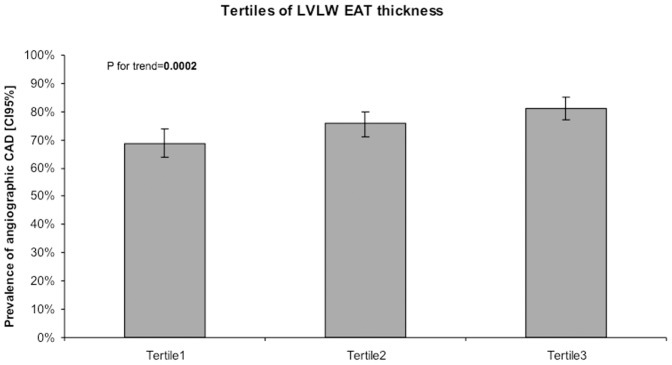
Association of angiographic coronary artery disease and left ventricle lateral wall (LVLW) epicardial adipose tissue (EAT) thickness tertile classification. CAD  =  coronary artery disease; LVLW  =  left ventricle lateral wall; EAT  =  epicardial adipose tissue. Prevalence of angiographic coronary artery disease and 95% confidence intervals [95% CIs] in LVLW EAT tertiles.

The probability of a stenosis on angiography increased with increasing LVLW EAT thickness. In the first tertile of EAT thickness, the prevalence of at least one significant coronary artery stenosis was 68.9% (n = 221). In the second and the third tertiles, the prevalence rose to 75.9% (n = 249) and 81.3% (n = 261), respectively (P for trend  = 0.0002) (Table 3 and Figure 1).

**Table 3:** Association between angiographic coronary artery disease and left ventricle lateral wall EAT thickness tertiles.

	Tertile1	Tertile2	Tertile3	P
Men; n (%)	230 (71.65%)	223 (67.99%)	236 (73.52%)	0.2861
Age (yrs.); mean ± SD	57.34±11.77	61.36±10.92	63.91±10.38	**<0.0001**
BMI; mean ± SD	26.51±4.73	27.74±4.50	27.90±4.19	**0.0001**
Current smoker; n (%)	108 (33.64%)	67 (20.43%)	69 (21.50%)	**0.0001**
Diabetes; n (%)	66 (20.56%)	76 (23.17%)	81 (25.23%)	0.3700
Hypertension; n (%)	144 (44.86%)	179 (54.57%)	178 (55.45%)	**0.0170**
Dyslipidemia; n (%)	146 (45.48%)	149 (45.43%)	132 (41.12%)	0.4412
Metabolic syndrome; n (%)	14 (4.36%)	28 (8.54%)	20 (6.23%)	0.0931
Calcium scoring; median [IQR]	6.0 [0.0; 180.0]	24.0 [0.0; 422.0]	37.0 [0.0; 406.0]	**0.0024**
Presence of angiographic CAD; n (%)	221 (68.85%)	249 (75.91%)	261 (81.31%)	**0.0012**

Abbreviations as in Table 1 and 2.

Simple linear regression analysis demonstrated that epicardial fat thickness was correlated with degree of coronary stenosis OR = 1.67, p = 0.0009 but not with waist circumference: OR = 1.01, p = 0.16 and BMI: OR = 1.02, p = 0.26 in CAD subjects. The independent relation of epicardial fat thickness, waist circumference and BMI with coronary stenosis was then assessed by a multiple regression analysis in CAD subjects. Epicardial fat thickness was the most significant independent correlate of degree of coronary stenosis, dependent variable in CAD subjects. (Table 4).

**Table 4:** Multivariable correlates of degree of coronary stenosis in CAD subjects.

	Univariate analysis			Multivariate analysis		
	OR	[95%CI]	P	OR	[95%CI]	P
BMI	1.02	[0.99–1.05]	0.2614	0.99	[0.92–1.06]	0.7239
Waist circumference	1.01	[0.99–1.03]	0.1604	1.01	[0.99–1.04]	0.3013
LVLW EAT (≥2.8 vs. 2.8 mm)	1.67	[1.23–2.26]	**0.0009**	1.67	[1.08–2.57]	**0.0197**

Abbreviations as in Table 1.

### Epicardial fat and extent of angiographic CAD

**Figure 2:**
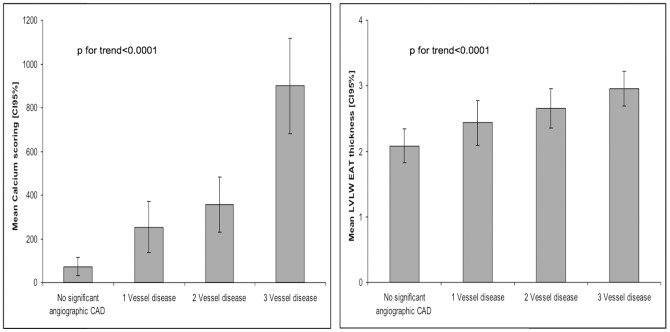
Association of left ventricle lateral wall (LVLW) epicardial adipose tissue (EAT) thickness and calcium scoring between patients with none or minimal coronary artery disease, 1, 2 or 3 vessel disease. Abbreviations as in Figure 1. Mean calcium scoring (Agatston score) and mean LVLW EAT thickness (mm) and 95% confidence intervals [95% CIs] in patients with no significant angiographic coronary artery disease, 1, 2 or 3 vessel disease.

Increased LVLW EAT thickness correlated positively with the severity or extent of CAD (Figure 2): LVLW EAT thickness mean was 2.08±2.1 mm for the patients with no or minimal vessel disease, 2.43±2.4 mm for the patients with single vessel disease, 2.65±2.2 mm for patients with 2 vessel disease or left main disease and 2.95±2.5 mm for patients with 3 vessels disease or left main + right coronary artery disease (p for trend = 0.0001).

Moreover, patients with multivessel disease had more often a LV wall EAT thickness>2.8 mm. 83 (34.73%) patients with no or minimal vessel disease had LVLW EAT thickness>2.8 mm and 177 (51.45%) patients with three vessels disease had a LVLW EAT thickness>2.8 mm. This relation was better with calcium scoring (Figure 2).

### Diagnostic performance of EAT and calcium scoring

**Figure 3:**
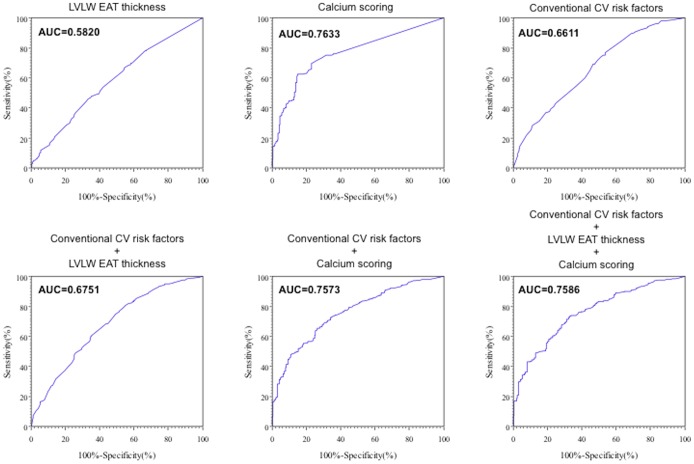
Receiver operating characteristic curves. Abbreviations as in Figure 1; CV =  cardiovascular.

On receiver operating characteristic (ROC) curve analysis, the best cut-off for LVLW EAT thickness to predict the presence of a significant coronary stenosis was 2.8 mm. LVLW EAT thickness ≥2.8 mm had a sensitivity of 46.1%, specificity of 66.5%, positive predictive value of 80%, and negative predictive value of 28.8%. The area under the curve was 0.58. The best cut-off for RVLW was 5.3 mm. RVLW EAT thickness ≥5.3 mm predicted the presence of significant coronary stenosis, with sensitivity of 45.1%, specificity of 67.9%, positive predictive value of 81.1%, and negative predictive value of 28.9%. The best cut-off for calcium scoring was 24 (Agatston score). Calcium scoring>24 predicted the presence of significant coronary stenosis, with sensitivity of 62.3%, specificity of 85.1%, positive predictive value of 89%, and negative predictive value of 54.2%. The area under the curve was 0.76 (Figure 3). Conventional cardiovascular risk factors ROC curve was determined and was added to LVLW EAT and calcium scoring measurement. Areas under the curve, sensibility, specificity, positive and negative predictive values are summarized in Table 5. LVLW EAT thickness and calcium scoring were statistically associated (p = 0.0125).

**Table 5:** Receiver operating characteristic curves: Areas under the curve, sensibility, specificity, positive and negative predictive values.

	AUC	Se	Sp	PPV	NPV
LVLW EAT thickness	0.5820	0.4610	0.6653	0.8081	0.2875
Calcium scoring	0.7633	0.6261	0.8514	0.8903	0.5418
Conventional CV risk factors	0.6611	0.7699	0.4635	0.8154	0.3956
Conventional CV risk factors + LVLW EAT thickness	0.6751	0.8061	0.4506	0.8187	0.4303
Conventional CV risk factors + calcium scoring	0.7573	0.6535	0.7352	0.8269	0.5230
Conventional CV risk factors + LVLW EAT thickness + calcium scoring	0.7586	0.7356	0.6706	0.8121	0.5672

AUC  =  area under the curve; Se  =  sensibility; Sp =  specificity; PPV  =  positive predictive value; NPV  =  negative predictive value; CV  =  cardiovascular; other abbreviations as in Table 1.

## Discussion

The present study sought to determine the link between EAT and CAD using data from the EVASCAN study [6], a large multicenter prospective study originally performed to establish the diagnostic accuracy of CTCA compared to conventional coronary angiography in a population of symptomatic patients with a clinical indication for anatomical coronary imaging.

This study confirmed that EAT thickness of left ventricle wall was associated with CAD and an independent predictor of CAD. Interestingly, we found that, even though mean right ventricle wall EAT was thicker than left ventricle wall EAT, EAT of the left ventricle but not the right ventricle wall was associated to CAD. Whether this association is driven by a functional or mechanical cause is unclear and further physiopathological studies need to be done.

Second, EAT thickness was well correlated to the severity of angiographic CAD. The greater the LVLW EAT thickness, the greater the probability of multivessel disease. Thirdly, EAT thickness was not associated with BMI and waist circumference and was the best predictor of CAD. Finally, when studying EAT as a diagnostic screening tool, it had poor performance, lower than calcium scoring. Adding EAT measurement to calcium scoring did not improve significantly the ROC curve.

There are two main implications of the present results. First, from a pathophysiologic perspective, the association between EAT and angiographic CAD is consistent with the hypothesis that epicardial fat may play a role in the genesis of CAD. EAT is the visceral fat depot of the heart. It is a metabolically active organ with anatomical and functional contiguity to the myocardium as it is located along the coronary arteries on the surface of the ventricles and the apex of the heart [10]. Because of its close proximity, epicardial fat can locally affect the heart and coronary arteries through vasocrine or paracrine secretion of a number of bioactive molecules and pro-inflammatory cytokines [11]–[13]. In fact, excess accumulation of epicardial fat was reported to be a stronger coronary risk factor than the distribution of other body fat [14]. On the basis of these findings, several clinical studies have investigated EAT as a cause of CAD, finding that EAT thickness and volume strongly correlated with atherosclerosis and coronary artery calcium score, both of which are characteristic of plaques and CAD [1]–[5]. Echocardiographic studies have suggested that EAT was neither strongly associated with the incidence of major adverse cardiovascular events in patients with CAD, nor associated with coronary artery stenosis [15]–[17]. More recently, a meta-analysis by Xu et al. [18], of 15 case–control studies and one case sectional study (N = 2872 patients) found a positive association between EAT thickness and volume and the presence of CAD. Our study confirms this association.

In addition to being associated with the presence of CAD, EAT has also been associated with fatal and nonfatal coronary events in the general population, independently of conventional cardiovascular risk factors [19]. As such, the presence of EAT may complement information from cardiac computed tomography over and above the CAC scoring. These findings are consistent with the observation that EAT is strongly associated not only with the presence, but also with the severity of CAD. Whether this association is driven by functional or mechanical causes is still unclear and further physiopathological studies with molecular insights for this relation are needed. While additional information on the biological profile of our patients, including biomarkers of inflammation, would have been useful they were not available since our data were obtained in routine medical care. Nevertheless, recent studies have shown that increased EAT thickness was associated with low grade systemic *inflammation* [20] and that orosomucoid secretion by EAT could be a possible indicator of endothelial dysfunction in diabetes mellitus [21]. Moreover, Hirata et al. showed that inflammatory cell infiltration was enhanced in EAT, but not in subcutaneous fat, in patients with CAD [22]. Chronic inflammation in epicardial fat may participate in the pathogenesis of coronary atherosclerosis and therefore, it may be interesting to combine EAT abundance and biological markers to improve the identification of patient at risk of CAD events.

We reported an association between EAT and calcium scoring. Whether EAT could influence calcium scoring is unclear. Previous studies have shown that EAT correlates with the extent of CAC [23]–[25]. Alexopoulos et al. [3] found that larger EAT volumes were associated with the presence of plaques with a non-calcified component. However that study had a wide range of EAT volumes in patients with mixed plaques. Nevertheless, this observation suggests that the release of noxious agents from EAT may sustain an active atherosclerotic process as proven by the presence of non-calcified plaques. The presence of mere CAC, instead, could represent a more advanced and stable phase of the atherosclerotic process. In support of this hypothesis, Broedl et al. [26]. found that low levels of adiponectin, which is secreted locally by EAT, were associated with the presence of non-calcified or mixed coronary plaques, but not with the presence of calcified plaques. These concepts still remain speculative. It should also be noted that Greif et al. [27] found no relationship between EAT and any type of atherosclerotic plaque. However, these investigators did not adjust for differences in risk factor prevalence, likely confounding their ability to detect an effect of EAT volume.

The second important implication is that, although EAT is strongly correlated to the presence and extent of angiographic CAD, it is probably of little value as a diagnostic or screening tool since other methods such as calcium scoring or computed tomography coronary angiography have far superior sensibility and specificity. Moreover, adding EAT measurements to calcium scoring did not improve the diagnostic performance.

### Limitations

The present study has some limitations. First, we measured EAT thickness rather than volume. However, even if multidetector CT measurements of volumetric EAT are considered more reproducible than multidetector CT measurements of EAT thickness [15], [28], EAT thickness is easier to perform and less time-consuming compared to the labor-intensive work for measurements of volumetric EAT and therefore more suitable for research purposes. Consequently, we measured EAT thickness on the left and right ventricle free wall because of its accessibility and potential for use in clinical practice. We noted a strong positive association between EAT thickness and coronary atherosclerosis quantified by CA. Patients underwent mostly 64 row cardiac CT (684 (70.5%) patients) and the other 286 patients (29.5%) had 16 to 40 row CT. Nevertheless, even if 16 row cardiac CT are not used anymore (patients were included between June 2006 and June 2008), it does not affect EAT thickness measurement reliability. Second, this study was performed in symptomatic patients and not in an asymptomatic population undergoing screening, which could influence the results of EAT as a screening tool. However, this study was first performed on a pathophysiologic approach, not to prove the accuracy of EAT thickness measurement as a screening tool in an asymptomatic cohort. Thirdly, despite the independent association between epicardial thickness and CAD, our study did not provide direct molecular insights into this relation. Fourth, coronary angiography alone is not the most accurate method to estimate coronary stenosis degree and severity, especially in early lesions, due to outward remodeling of coronary vessels. However, coronary angiography represents the most widely used technique for assessing coronary anatomy. In the literature, significant stenosis is defined as>50% stenosis of the luminal diameter but this does not account for the presence of plaque instability, which may lead to acute coronary artery stenosis. Finally, we measured EAT in different parts of the heart, but the fact that it was not measured in right atrioventricular groove, left atrioventricular groove and interventricular groove could be a limitation.

## Conclusions

From a pathophysiologic perspective, our study demonstrated a strong association between EAT and the presence and extent of angiographic CAD. It is consistent with the hypothesis that epicardial fat may play a role in the genesis of CAD, possibly due to paracrine or vasocrine mechanisms. Although EAT is correlated to CAD, it is probably of little value as a diagnostic or screening tool since other methods such as calcium scoring or computed tomography coronary angiography have far superior sensibility and specificity.

## References

[1] Wang TD, Lee WJ, Shih FY, Huang CH, Chen WJ, et al (2010) Association of epicardial adipose tissue with coronary atherosclerosis is region-specific and independent of conventional risk factors and intra-abdominal adiposity. Atherosclerosis 213(1): 279–87. 20801451 10.1016/j.atherosclerosis.2010.07.055

[2] Bettencourt N, Toschke AM, Leite D, Rocha J, Carvalho M, et al (2012) Epicardial adipose tissue is an independent predictor of coronary atherosclerotic burden. Int J Cardiol 28 158(1): 26–32.. 21255849 10.1016/j.ijcard.2010.12.085

[3] Alexopoulos N, McLean DS, Janik M, Arepalli CD, Stillman AE, et al (2010) Epicardial adipose tissue and coronary artery plaque characteristics. Atherosclerosis 210(1): 150–4.. 20031133 10.1016/j.atherosclerosis.2009.11.020

[4] Oka T, Yamamoto H, Ohashi N, Kitagawa T, Kunita E, et al (2012) Association between epicardial adipose tissue volume and characteristics of non-calcified plaques assessed by coronary computed tomographic angiography. Int J Cardiol 161(1): 45–9.. 21570136 10.1016/j.ijcard.2011.04.021

[5] Eroglu S, Sade LE, Yildirir A, Bal U, Ozgul AS, et al (2009) Epicardial adipose tissue thickness by echocardiography is a marker for the presence and severity of coronary artery disease. Nutr Metab Cardiovasc Dis 19(3): 211–7.. 18718744 10.1016/j.numecd.2008.05.002

[6] Gueret P, Deux JF, Bonello L, Sarran A, Tron C, et al (2013) Diagnostic Performance of Computed Tomography Coronary Angiography (from the Prospective National Multicenter Multivendor EVASCAN Study). Am J Cardiol 111(4): 471–8. 23261002 10.1016/j.amjcard.2012.10.029

[7] Menzel HG, Shibilla H, Teunen D, editors (2000) European guidelines on quality criteria for computed tomography. Luxembourg: European Commission Publication. (No. EUR 16262 EN. 2000).

[8] Smith SC, Feldman TE, Hirshfeld JW, Jacobs AK, Kern MJ, et al (2006) ACC/AHA/SCAI 2005 guidelines update for percutaneous coronary intervention—summary article: a report of the American College of Cardiology/American Heart Association Task Force on Practice Guidelines (ACC/AHA/SCAI Writing Committee to Update the 2001 Guidelines for Percutaneous Coronary Intervention). J Am Coll Cardiol 47(1): 216–35. 16386696 10.1016/j.jacc.2005.11.025

[9] Austen WG, Edwards JE, Frye RL, Gensini GG, Gott VL, et al. (1975) A reporting system on patients evaluated for coronary artery disease. Report of the Ad Hoc Committee for Grading of Coronary Artery Disease, Council on Cardiovascular Surgery, American Heart Association. Circulation 51 (4 Suppl):5–40. 10.1161/01.cir.51.4.5 1116248

[10] Sacks HS, Fain JN (2007) Human epicardial adipose tissue: A review. Am Heart J 153(6): 907–17.. 17540190 10.1016/j.ahj.2007.03.019

[11] Baker AR, Silva NF, Quinn DW, Harte AL, Pagano D, et al (2006) Human epicardial adipose tissue expresses a pathogenic profile of adipocytokines in patients with cardiovascular disease. Cardiovasc Diabetol 13 5: 1.. 16412224 10.1186/1475-2840-5-1 PMC1352345

[12] Rabkin SW (2007) Epicardial fat: properties, function and relationship to obesity. Obes Rev 8(3): 253–61.. 17444966 10.1111/j.1467-789X.2006.00293.x

[13] Mazurek T, Zhang L, Zalewski A, Mannion JD, Diehl JT, et al (2003) Human epicardial adipose tissue is a source of inflammatory mediators. Circulation 108(20): 2460–6.. 14581396 10.1161/01.CIR.0000099542.57313.C5

[14] Taguchi R, Takasu J, Itani Y, Yamamoto R, Yokoyama K, et al (2001) Pericardial fat accumulation in men as a risk factor for coronary artery disease. Atherosclerosis 157(1): 203–9.. 11427222 10.1016/s0021-9150(00)00709-7

[15] Chaowalit N, Somers VK, Pellikka PA, Rihal CS, Lopez-Jimenez F (2006) Subepicardial adipose tissue and the presence and severity of coronary artery disease. Atherosclerosis 186(2): 354–9.. 16183065 10.1016/j.atherosclerosis.2005.08.004

[16] Albuquerque FN, Somers VK, Blume G, Miranda W, Korenfeld Y, et al (2012) Usefulness of epicardial adipose tissue as a predictor of adverse cardiovascular events in patients with coronary artery disease. Am J Cardiol 110(8): 1100–5.. 22762719 10.1016/j.amjcard.2012.06.003

[17] Parcon R, Kruk M, Kepka C, Pregowski J, Opolski MP, et al (2011) Epicardial adipose tissue radiodensity is independently related to coronary atherosclerosis. A multidetector computed tomography study. Circ J 75(2): 391–7.. 21178296 10.1253/circj.cj-10-0441

[18] Xu Y, Cheng X, Hong K, Huang C, Wan L (2012) How to interpret epicardial adipose tissue as a cause of coronary artery disease: a meta-analysis. Coron Artery Dis 23(4): 227–33.. 22361934 10.1097/MCA.0b013e328351ab2c

[19] Mahabadi A, Berg M, Lehmann N, Kälsch H, Bauer M, et al (2013) Association of Epicardial Fat With Cardiovascular Risk Factors and Incident Myocardial Infarction in the General Population: The Heinz Nixdorf Recall Study. J Am Coll Cardiol 61(13): 1388–95.. 23433560 10.1016/j.jacc.2012.11.062

[20] Tok D, Kadife I, Turak O, Ozcan F, Basar N, et al (2012) Increased epicardial fat thickness is associated with low grade systemic inflammation in metabolic syndrome. Turk Kardiyol Dern Ars 40(8): 690–5.. 23518882 10.5543/tkda.2012.60207

[21] Fandiño-Vaquero R, Fernández-Trasancos A, Alvarez E, Ahmad S, Batista-Oliveira AL, et al (2014) Orosomucoid secretion levels by epicardial adipose tissue as possible indicator of endothelial dysfunction in diabetes mellitus or inflammation in coronary artery disease. Atherosclerosis 235(2): 281–288.. 24905138 10.1016/j.atherosclerosis.2014.05.921

[22] Hirata Y, Kurobe H, Akaike M, Chikugo F, Hori T, et al (2011) Enhanced inflammation in epicardial fat in patients with coronary artery disease. Int Heart J 52(3): 139–42.. 21646734 10.1536/ihj.52.139

[23] Rosito GA, Massaro JM, Hoffmann U, Ruberg FL, Mahabadi AA, et al (2008) Pericardial fat, visceral abdominal fat, cardiovascular disease risk factors, and vascular calcification in a community-based sample: the Framingham Heart Study. Circulation 117: 605–13. 18212276 10.1161/CIRCULATIONAHA.107.743062

[24] de Vos AM, Prokop M, Roos CJ, Mejis MF, van der Schouw YT, et al (2008) Peri-coronary epicardial adipose tissue is related to cardiovascular risk factors and coronary artery calcification in post-menopausal women. Eur Heart J 29: 777–83. 18156138 10.1093/eurheartj/ehm564

[25] Ding J, Kritchevsky SB, Harris TB, Burke GL, Detrano RC, et al (2008) The association of pericardial fat with calcified coronary plaque. Obesity (Silver Spring) 16: 1914–9. 18535554 10.1038/oby.2008.278 PMC4098129

[26] Broedl UC, Lebherz C, Lehrke M, Stark R, Greif M, et al (2009) Low adiponectin levels are an independent predictor of mixed and non-calcified coronary atherosclerotic plaques. PLoS One 4: e4733. 19266101 10.1371/journal.pone.0004733 PMC2649379

[27] Greif M, Becker A, von Ziegler F, Lebherz C, Lehrke M, et al (2009) Pericardial adipose tissue determined by dual source CT is a risk factor for coronary atherosclerosis. Arterioscler Thromb Vasc Biol 29: 781–6. 19229071 10.1161/ATVBAHA.108.180653

[28] Gorter PM, van Lindert AS, de Vos AM, Mejis MF, van der Graaf Y, et al (2008) Quantification of epicardial and peri-coronary fat using cardiac computed tomography; reproducibility and relation with obesity and metabolic syndrome in patients suspected of coronary artery disease. Atherosclerosis 197(2): 896–903.. 17884060 10.1016/j.atherosclerosis.2007.08.016

